# Rapid mobile inspection equipment for metro tunnels based on multi-sensor integration

**DOI:** 10.1038/s41598-025-18959-y

**Published:** 2025-10-07

**Authors:** Tingli Fan, Qingzhou Mao, Chao Tang, Li Wang, Jiulin Li, Yan Bao, Tao Gao, Lejun Pei, Dongliang Zhang, Yong Wang

**Affiliations:** 1 School of Remote Sensing Information Engineering, Wuhan University, Wuhan, China; 2 Engineering Research Center for Spatial-Temporal Data Smart Acquisition and Application, Ministry of Education of China, Wuhan, China; 3 Beijing Urban Construction Exploration & Surveying Design Research Institute CO, LTD, Beijing, China; 4 The Key Laboratory of Urban Security and Disaster Engineering of China, Ministry of Education, Beijing University of Technology, Beijing, China; 5 Beijing Urban Construction Group Co., Ltd., Beijing, China; 6 School of Future cities, University of Science and Technology Beijing‌, Beijing, China

**Keywords:** Metro tunnel, Laser scanning system, CCD cameras, 3D modeling, Defects detection, Civil engineering, Electrical and electronic engineering

## Abstract

The acquisition of tunnel inspection data is fundamental to tunnel operation and maintenance. Existing inspection equipment is either based on laser scanning or CCD cameras. Laser scanning-based devices often struggle to detect cracks, while equipment based on CCD cameras is unable to acquire point cloud to assess tunnel deformations. In order to obtain high-quality, comprehensive data, this paper develops a mobile three-dimensional inspection device CKY-200, integrated with multiple sensors. The CKY-200 addresses the temporal and spatial synchronization issues of CCD cameras and laser scanners. Considering the convenience of inspection, the device progresses on the track powered by electricity. Moreover. This article proposes a tunnel deformation detection algorithm and a crack width measurement method, and verifies the accuracy of the data collected by the equipment. Through on-site experiments and comparison, the CKY-200 provides the advantages of more comprehensive data acquisition while ensuring high precision and speed.

## Introduction

The development of urban areas has driven the widespread construction of metros. Researches indicate that during their prolonged usage, tunnel structures undergo changes in their stress states due to factors such as surrounding soil and groundwater. These changes inevitably result in subway tunnels experiencing issues such as deformation and cracking^1,2^. These issues pose a severe threat to public life and property safety and can impact the development of urban infrastructure. Therefore, it is essential to regularly monitor the health status of the tunnels, comprehensively understand the current condition of subway defects.

Traditional tunnel inspection methods involve manual visual inspections combined with various measuring instruments. In recent years, the development of modern surveying and computer technology has introduced new ideas and approaches for defects detection. Experts have conducted significant researches^3–7^ in tunnel inspection equipment in airborne, vehicle-mounted and ground three-dimensional measurements, obtaining notable progress. This includes the use of drones, inspection vehicles, and inspection robots.

Unlike other inspection tasks, metro tunnel detection is restricted to a specific time during the night. The length of subway tunnel sections primarily ranges between 1.2 and 2 km. Disregarding the preparation time, in traditional manual visual inspections, it approximately takes 1.5 h each night to use equipment for inspecting 2–3 sections^8^. Therefore, small-scale inspection vehicles, characterized by their modular design, compactness, and rapid assembly features, greatly facilitate on-site inspection processes. Mature products include the ATLAS70^9^ tunnel detection system and the GPR5000 tunnel surface monitoring system^10^, etc. The GPR5000 is a hand-pushed rail cart, which itself lacks autonomous operation capabilities, and is equipped with a three-dimensional laser sensor. To ensure the accuracy of the GPR5000 detection results, it is specified that the operating speed should be controlled within 0.7 km/h. Therefore, the detection speed of this equipment is relatively slow. The FAST^11^ utilizes a vehicle-mounted ultra-high frequency planar array camera, employing fixed-focus industrial cameras in eight or more groups to achieve full cross-sectional imaging of tunnels. The system’s highest resolution is 0.2 × 0.2 mm, and the maximum detection speed is 50 km/h, capable of identify cracks wider than 0.3 mm. The Swiss company Terra has developed the tCrack system^12^, which is equipped with multiple line-scan cameras. It can be mounted on rail or road vehicles. At a collection speed of 2.5 km/h, the system can achieve a crack detection precision of up to 0.3 mm. Xiaojun Wu and colleagues^13^ have developed an image acquisition system based on CMOS linear array cameras. This system is equipped with nine CMOS linear array industrial cameras, using red lasers as the light source. The detection vehicle is pulled by one or two rail cars, with the camera and light source system mounted on the exterior of the train’s trail, while other equipment is installed inside the carriage. The system is capable of detecting cracks as small as 0.21 mm. Xuanran Liu^14^ from Beijing Jiaotong University has designed a self-powered tunnel inspection vehicle. This inspection vehicle is equipped with array cameras, lighting, and other equipment. It can inspect at speeds up to 30 km/h, with a detection accuracy of 0.2 millimeters per pixel. Hongwei Huang and his team at Tongji University have introduced the MTI-200a^15^, a metro tunnel disease detection device. This device is equipped with six linear array cameras with a resolution of 7.5 k, covering approximately 290°of the circular tunnel. It can detect with an accuracy of 0.2 millimeters per pixel and can inspect at a maximum speed of 10 km/h.

It is evident that the existing visual object detection and laser scanning and detection in a point cloud technology have both achieved a high level of detection accuracy, capable of acquiring high-quality image data or laser point cloud data. However, these technologies have some limitations: The single measurement detection functionality and the detection vehicles based on laser scanning often cannot detect cracks due to the limitations of the laser scanners themselves. Meanwhile, detection vehicles equipped with digital photography can only detect cracks and cannot acquire point cloud data, making it impossible to detect tunnel deformations. Additionally, these devices are unable to collect more types of data within a limited time frame^16^. Therefore, their comprehensive capabilities need to be improved.

This paper develops a mobile inspection device CKY-200, It is used to collect tunnel surface image data and point cloud data. The proposed device realises the temporal and spatial synchronisation of CCD (Charge Coupled Device) cameras and a laser scanner, which can simultaneously collect point cloud data and image data in one inspection work. Additionally, an experiment is carried out in Wuhan metro tunnel to verify the feasibility and accuracy of the equipment.

The remainder of this paper is arranged as follows. Section 2 introduces the design of CKY-200 and the working principle of the main parts as well as the time synchronisation technology between the sensors. Section 3 introduces the data processing algorithms for the images and point clouds acquired by CKY-200. Section 4 introduces the experiment process and data processing results of this paper. Finally, Sect. 5 presents the conclusion of this study.

## The design parameters for CKY-200

The detection task inside the tunnel involves using mobile sensors to collect information about the lining surface at a certain speed. The most widely used machine vision sensors currently are Charged-Coupled Device (CCD) cameras or laser scanners. However, since existing sensors cannot capture both point cloud data and image data of the tunnel simultaneously, there is a need to redesign the machine vision system. This redesign aims to enrich the types of data captured, while also enhancing the accuracy and efficiency of detection. This section discusses the main parameters such as equipment hardware design and detection speed.

### Overview of the equipment

**Fig. 1:**
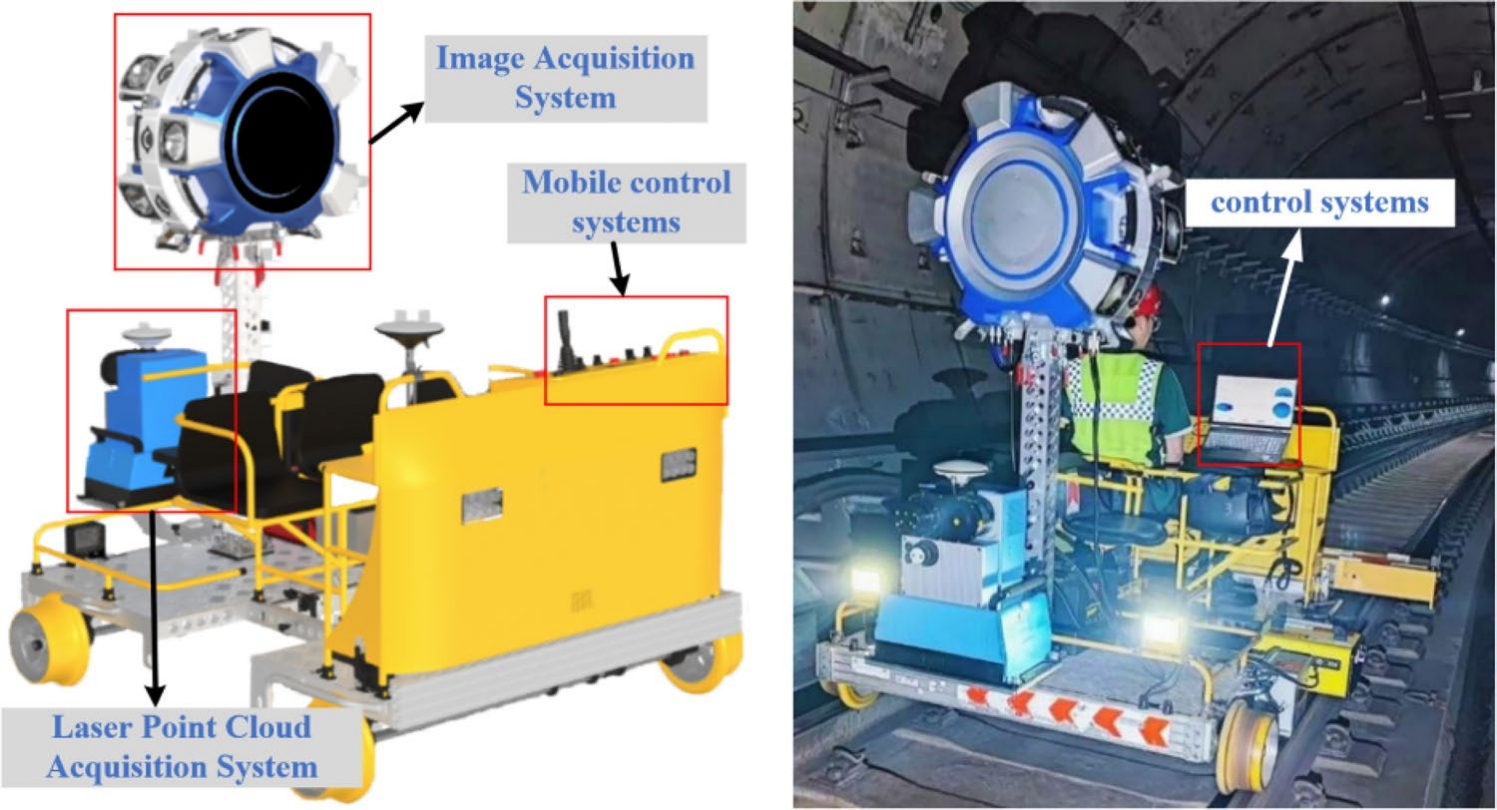
Blueprint and prototype image of the entire system.

This equipment is designed for comprehensive inspection of metro tunnels, suitable for various types of metro tunnels, including circular shield tunnels and horseshoe-shaped tunnels, and is compatible with track gauges of approximately 1435 ± 20 mm. The device is an electrically driven, vehicle-mounted intelligent tunnel structure detection system. As shown in Fig. 1, the detection equipment highly integrates high-precision laser scanners, CCD panoramic cameras, GNSS (Global Navigation Satellite System)/IMU (Inertial Measurement Unit) (high precision laser inertial navigation)/DMI (Distance measurement indicator) combined positioning and attitude sensors, as well as control and storage sensors. It mainly consists of four systems: the point cloud collection system, image collection system, mobile control system and control system. As shown in Fig. 1, these systems are respectively responsible for point cloud data collection, image collection, equipment movement, and data synchronization, in order to acquire high-quality 3D point cloud data of tunnels, high-definition images, and accurate mileage location information.

**Fig. 2:**
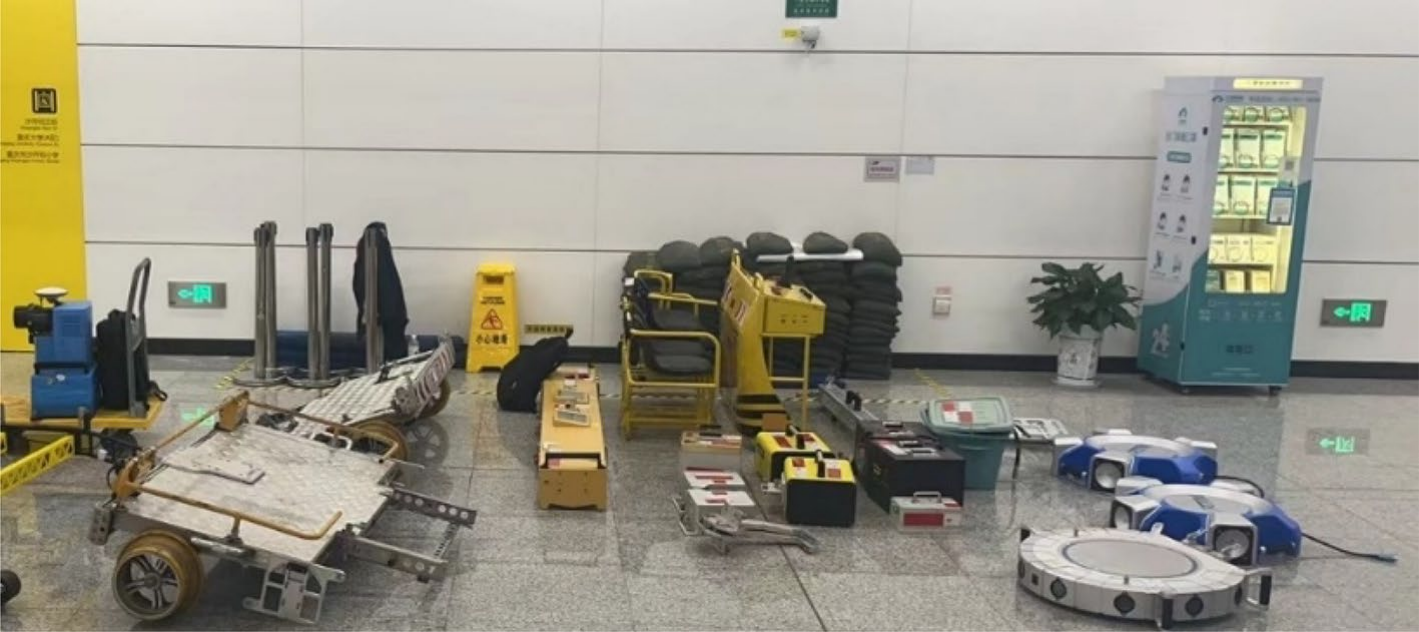
Detachable device.

The equipment progresses on the track surface with its own electrically driven power, eliminating the need for manual pushing, and can carry 3–4 inspection personnel with a load capacity of more than or equal to 500 kg. As shown in Fig. 2, it consists of parts such as the front bracket, rear bracket, control console, and seats. Since the inspection equipment cannot be left inside the subway tunnel, it must be moved out of the tunnel after inspection and moved into the tunnel before inspection. Therefore, to save actual inspection time, the equipment is designed to be detachable, allowing for quick assembly and disassembly. Both the overall structure weight and the individual unit weight have been optimized to ensure that 2–4 people can carry and assemble it on the track. Moreover, the equipment supports a constant speed cruising feature.

### 3D laser scanning detection module

Tunnel convergence deformation is an important indicator reflecting the tunnel load bearing situation and the tunnel service performance. Existing research show that tunnel convergence detection results obtained through calculations using three-dimensional laser point cloud data have high accuracy^17^. Therefore, the laser scanning detection module is considered an important detection module.

The hardware system of the laser scanning detection module adopts a pixel-by-pixel scanning method. After numerous simulation tests, it can use external signals to calculate the position of scanning data. In order to achieve high-precision scanning of high-density cross-sections, the PROFILER 9012 was selected as the laser sensor. The PROFILER 9012, based on the IMAGER 5010 and a 2D cross-sectional laser measurement system, is a scanner developed to scan objects within a distance of up to 119 m. Considering that the diameter of tunnels usually does not exceed 7 m, the PROFILER 9012 can completely cover the detection range of the tunnel, ensuring the comprehensiveness and accuracy of the data.

**Fig. 3:**
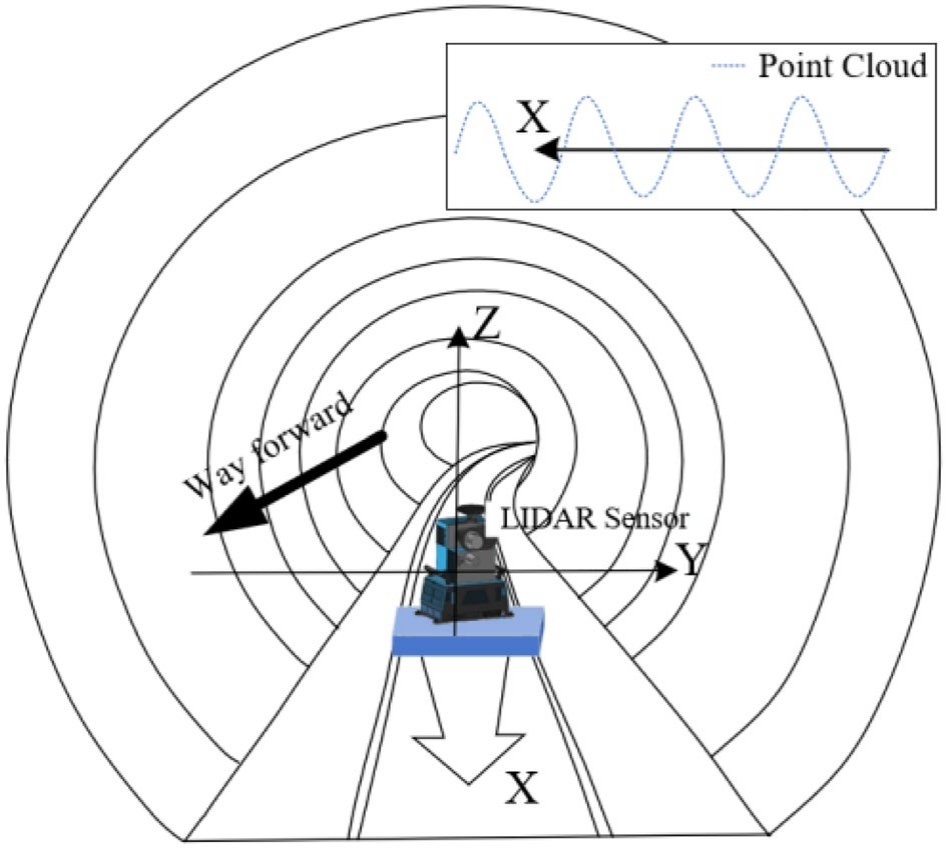
The working principle of a mobile 3D laser measurement system in tunnel.

As shown in Fig. 3, the laser scanner is mounted on a mobile device and operates in line scanning mode while the device advances. During the progression of the device, the laser beam remains perpendicular to the direction of the device’s movement. The laser emitter rotates 360° vertically, emitting laser beams at a high frequency towards the target being measured. The sensor receives the reflected laser signals and records the time difference (phase difference). By analyzing the emitted and received laser signals (intensity and phase difference), point cloud data of the inner surface of the tunnel lining (along the Z and Y axes) are recorded. Additionally, by combining this data with the X-axis (direction of the tunnel axis) mileage information recorded by the odometer on the rail cart, these data are temporally and spatially calibrated and stitched together to ultimately produce a complete 3D point cloud dataset.

In the process of advancing along the tunnel, the rotating axis of the scanner in the mobile 3D scanning system always remains parallel to the tangent of the tunnel’s central axis. This alignment ensures that the laser’s incidence angle remains nearly perpendicular to the tunnel walls. Benefiting from a point cloud scanning speed of over one million points per second and a cross-sectional scanning speed of up to 200 revolutions per second, up to 20,000 sample points can be collected in each rotational period. To ensure the quality of the point cloud, the cart’s forward speed is limited to less than 15 km/h. Since the rotational speed of the laser emitter is much greater than the cart’s forward speed, the point cloud obtained from one complete rotation of the laser can be regarded as being on the same cross-sectional plane of the tunnel, making the error in the direction of the tunnel’s axis negligible. When the cart moves at a constant speed, the intervals between each column of points are uniform. The density of points in the X-axis direction is positively correlated with the speed of the cart, thereby achieving high-precision scanning of high-density cross-sections.

### CCD cameras part

**Fig. 4:**
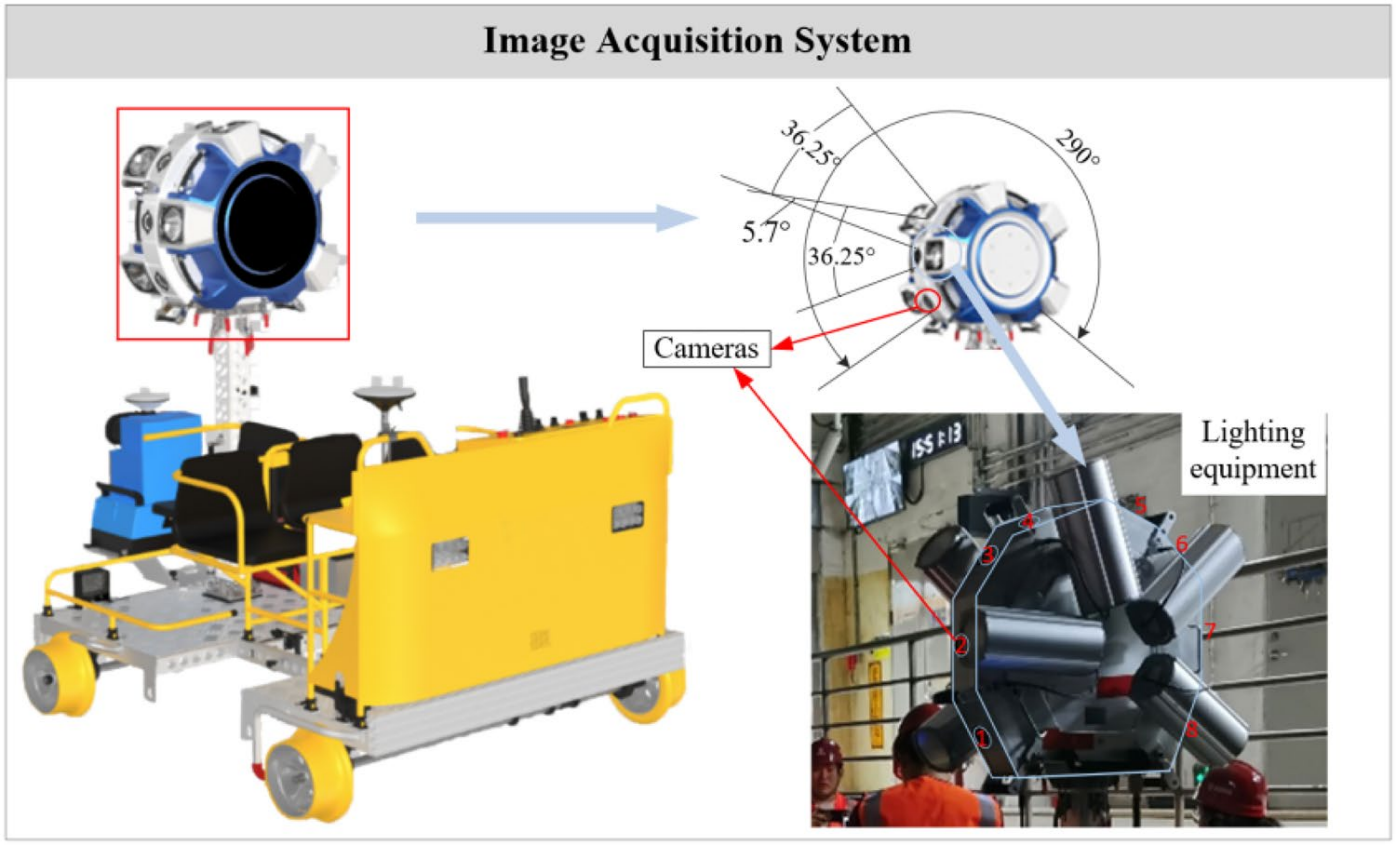
Subsystem-cameras with supplementary lighting equipment system.

The camera system employs Basler’s acA4096-30 μm area array cameras, which are equipped with Sony IMX 267 CMOS sensors. These cameras offer a frame rate of up to 32 fps and a resolution of 8.9 million pixels, enabling the device to detect even narrow hairline cracks. As shown in Fig. 4, the cameras’ horizontal and vertical field of view (FOV) angles are 47.65ºand 26.31º, respectively, with a single effective camera FOV of 36.25º. The overlapping angle between adjacent cameras is 5.70º, resulting in an overlap length of 0.23 m. To capture comprehensive tunnel information, the tunnel image acquisition subsystem integrates eight sets of industrial cameras and flash units, achieving an overall shooting angle of 290°. Ultimately, this system covers the tunnel lining sections that need inspection, excluding the bottom track bed, thereby meeting the tunnel inspection requirements.

**Fig. 5:**
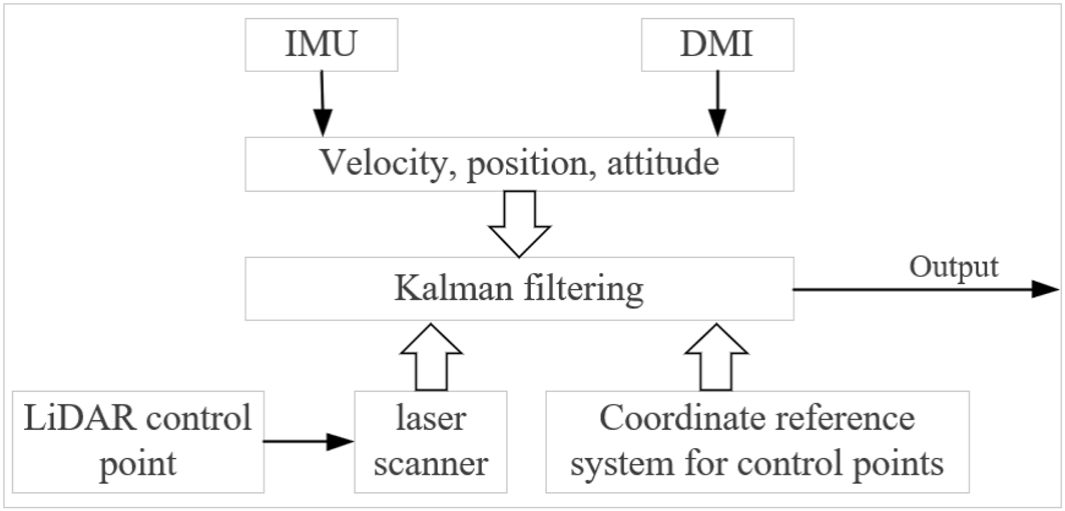
LiDAR/IMU/DMI combination algorithm.

As a foundational step for subsequent processes, it is crucial to capture images in a stable and clear manner. Unlike surface environments, underground tunnels lack natural lighting, making it essential to employ illumination devices to enhance the image capture of tunnel lining surfaces. As shown in the Fig. 5, each high-definition industrial camera is integrated with high-frequency supplementary lighting equipment. These devices provide ample lighting for the cameras, supporting their normal operation in day-night cycles and within tunnels. This ensures the system functions properly during rapid changes in brightness, such as when the inspection vehicle enters or exits the tunnel. The designed detection equipment can achieve a resolution of 2048 × 4098 pixels.

### Geometry part

The data acquisition parts were discussed in Sect. 2.2 and Sect. 2.3. This section elaborates on the sensors used for acquiring point cloud and image data’s positioning information, which will serve as the foundation for data processing.

**Fig. 6:**
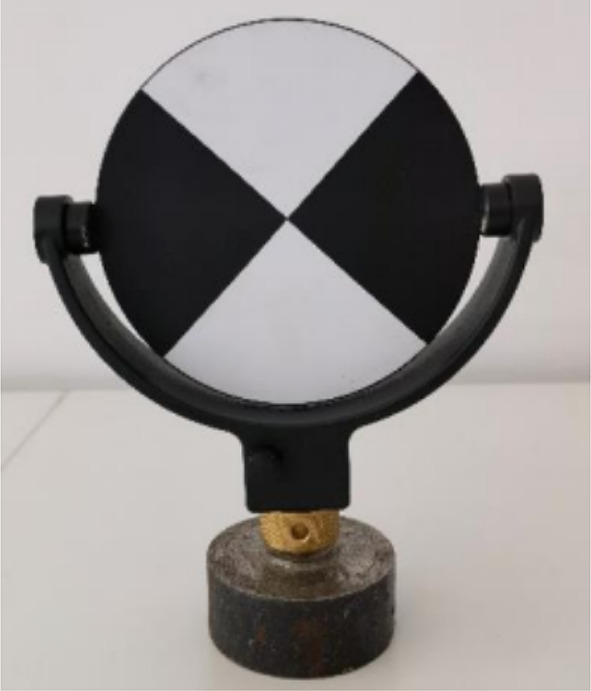
Schematic diagram of target points.

The Geometry section is primarily concerned with the positioning and orientation of the collection system, including GNSS receivers, Inertial Measurement Units (IMU), and Distance Measuring Instrument (DMI). The main purpose of the GNSS receiver is to expand the use cases of the equipment, so that it is not limited to underground use only. When GNSS signals are available, the GNSS data provides WGS 84 coordinate data for more convenient operation. In scenarios without GNSS conditions, the IMU and odometer are used as the core sensors of the POS, as shown in Fig. 5. Due to the absence of GNSS signals in metro tunnel environments, external reference information to correct positioning errors is unavailable, and the coordinate systems of the observed data cannot be unified. As a result, relying solely on IMU data leads to changes in the trends and randomness of errors. Consequently, this device offers an IMU/DMI combined positioning and orientation system suitable for environments lacking GNSS signals, aiming to overcome the limitations of single navigation technologies. By replacing the prism of the CPIII control point with a target as the laser scanner control points, The real coordinate data of CPIII is incorporated into Kalman filter equation, incorporating the laser scanner control point target data into the Kalman filtering equation, the error state vector of the IMU/DMI is calculated to limit the error dispersion to obtain high accuracy position and attitude, as shown in Fig. 6.

In a mobile 3D laser scanning system, the Inertial Measurement Unit (IMU) serves as the core component for acquiring absolute data. The IMU is a TYL50A product from China’s Huazhong Tianyixing Inertia Technology Co. It is utilized for measuring the three-axis attitude angles (or angular velocity) and acceleration of a rail trolley. The IMU primarily consists of three accelerometers and three gyroscopes. The accelerometers are responsible for measuring the linear motion of the carrier relative to inertial space, directly outputting specific force information, defined as the vector difference between the carrier’s absolute acceleration in inertial space and gravitational acceleration. Gyroscopes, on the other hand, are used for measuring the carrier’s angular motion relative to inertial space, directly outputting angular increment or angular velocity information. The inertial navigation system operates at a measurement frequency of 1000 Hz, with a drift of 0.02°per hour, and achieves a measurement precision of 1 mm over a 150 m chord length.

The three accelerometers separately integrate the velocity in each of the three directions, which allows the determination of the instantaneous position $\:{\:t}_{k}$of the moving object at a given time stamp $\:t$. This can be represented by the Eq. (2−1):

2-1 $$\:r\left({t}_{k}\right)=r\left({t}_{0}+{\int\:}_{0}^{k}v\left(t\right)dt\right)$$

In this equation, $\:{t}_{0}$ represents the position vector of the object at the initial time stamp. The principle of velocity measurement in each direction is explained in Eq. (2–2). Here, $\:v$ is the velocity at time $\:t$, $\:v\left({t}_{0}\right)$ is the initial velocity vector of the object, and $\:a$ is the acceleration.

2-2 $$\:v\left({t}_{k}\right)=v\left({t}_{0}\right)+{\int\:}_{0}^{k}a\left(t\right)dt$$

The DMI (Distance Measurement Indicator) is primarily used to record the distance traveled by a device, thereby providing real-time distance information during the movement of the entire system. This information lays the foundation for the computation of the position and orientation data for various modules of the system. In this study, we use the TRD-2TH1000V type odometer from GuangYang Electronics (Wuxi, China) Co., Ltd., which has a pulse frequency of up to 3600 lines/s and a distance resolution of up to 1 mm. During the device’s movement, the odometer is connected to the single wheel of the device. The rotation of the driving wheel drives the roller of the odometer, thus recording the traveled distance. The number of wheel rotations $\:N$, can be represented by Formula (2–3). In this formula, the parameter represents the number of counts obtained per revolution of the measuring wheel of the odometer. $\:{c}_{1}$ denotes the number of pulses at the termination of the wheel, $\:{c}_{2}$ denotes the number of pulses at the start of the wheel, and n denotes the number of pulses corresponding to one revolution.

2-3 $$\:N=\frac{{c}_{1}-{c}_{2}}{n}$$

The distance $\:S$ traveled by the cart can be calculated using the Eq. (2–4), where$\:\:r$ is the radius of the rail cart’s wheel:

2-4 $$\:S=2Nr\pi\:$$

The IMU’s center point is used as the origin of the carrier platform’s coordinate system. Once the device is assembled, the inertial guide must remain stationary and motionless for approximately 3 min, during which time the inertial guide collects data thousands of times as its starting point to ensure that the initial position is accurate. It then moves forward again to collect data. At the end, it is stationary again for another three minutes of acquisition. Errors during this process are corrected by the LIDAR control point coordinates and algorithms. The relationship between the residual observation $\:{\varDelta\:X}^{g}$ at the LiDAR control point and the position attitude error [$\Delta P,{\text{~}}\Delta o$] is given by Eq. (2–5):

2-5 $$\Delta X^{g} = \Delta P + B_{o} \Delta o + ~\varepsilon$$

Note that when extracting control point centroids from a point cloud, there may be small deviations, mainly caused by positioning errors. Let X be the IMU error state vector and the observation $\:z={\varDelta\:X}^{g}$, then the observation equation of the system can be expressed as Eq. (2–6), where $H = \left[ {I_{{3 \times 3}} ,0_{{3 \times 3}} } \right]$.

2-6 $$\:z = HX + \:\varepsilon$$

Based on the IMU dynamics model and the error model, the LiDAR control point data and the IMU and DMI data are entered into the Kalman filter equation. The error state vector of the IMU/DMI is calculated to limit its error dispersion^18–21^. The Kalman filtering process is as follows:

Time update:

2-7 $$\:{X}_{k+1|k}^{{\prime\:}{\prime\:}}=(1+{F}^{{\prime\:}{\prime\:}}\varDelta\:t){X}_{k}^{{\prime\:}{\prime\:}}$$

2-8 $$\:{D}_{k+1|k}^{{\prime\:}{\prime\:}}=(1+{F}^{{\prime\:}{\prime\:}}\varDelta\:t){D}_{k}^{{\prime\:}{\prime\:}}{(1+{F}^{{\prime\:}{\prime\:}}\varDelta\:t)}_{K}^{T}+Q$$

Status updates:

2-9 $$\:{X}_{k+1}^{{\prime\:}{\prime\:}}={X}_{k+1|k}^{{\prime\:}{\prime\:}}+{X}_{k+1}^{{\prime\:}{\prime\:}}({z}_{k}-H{X}_{k+1|k}^{{\prime\:}{\prime\:}})$$

2-10 $$\:{X}_{k+1}={D}_{k+1|k}{H}^{T}{(H{P}_{k+1|k}{H}^{T}+R)}^{-1}$$

2-11 $$\:{D}_{k+1}^{{\prime\:}{\prime\:}}=(I-{K}_{k+1}H){XD}_{k+1|k}^{{\prime\:}{\prime\:}}$$

where $\:{X}^{{\prime\:}{\prime\:}}$is the state vector without position error,$\:\:Q$s the equivalent system noise matrix, $\:H$is the observation matrix,$\:P$is the 3D position, and $\:R$ is the observation noise covariance matrix. $\:z$ is calculated as in Eq. (2–12):

2-12 $$z = \Delta V\delta t - \Delta P_{s} \Delta o - M\Delta S + \varepsilon$$

where, $\:\varDelta\:V$ is the velocity error correction vector, $\:\delta\:t$is the filter period, $\:\varDelta\:{P}_{s}$ is the IMU and odometer derived position increment, $\Delta o$ is the attitude error correction vector, $\:M$is the sparse matrix of odometer parameter error vectors, $\:\varDelta\:S$ is the odometer parameter error correction vector, and $\:\epsilon\:$is the equivalent observation noise. After obtaining the velocity error correction amount, the position error correction amount can be obtained by multiplying by the filter period.

The sources of interference in the data acquisition process are mainly electromagnetic radiation interference and environmental and structural interference. Electromagnetic radiation interference will lead to the expansion of the position solving error of the IMU after a long time of operation, and the point cloud aberration will cause a decrease in the deformation accuracy; the humid environment in the tunnel leads to a decrease in the insulation resistance, which triggers the leakage current interference. As the acquisition time of the device is from 0:00 a.m. to 4:00 a.m., when the tunnel needs to be powered off, the influence of electromagnetic interference is small, and the LIDAR circuit board is wrapped with copper foil to reduce the interference of electromagnetic radiation. The overall protection level of the device is IP54, and the key interface adopts aviation socket and filled with silicone sealing ring to effectively prevent the interference of the environment.2.5 Multi-sensor spatio-temporal synchronization.

In the measurement system, each sensor collects data according to its own sampling interval, leading to different input frequencies and varying levels of time precision. To correlate data measured by various sensors at the same time stamp and thus facilitate the integration and processing of multi-source data, it is imperative that the data collected by all sensors in the system are aligned on the same time axis, enabling the integration of any data.

**Fig. 7:**
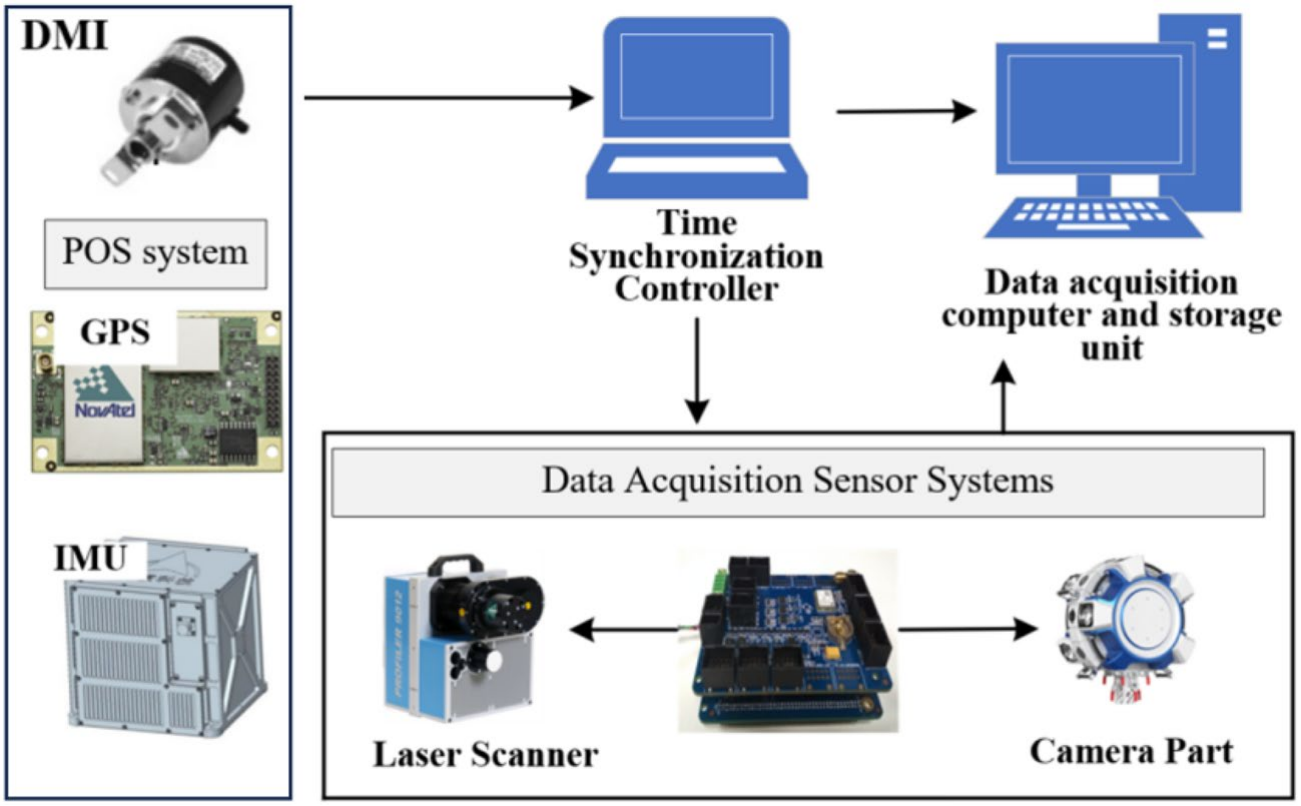
The synchronization control system.

The synchronization control system of the mobile 3D laser scanning measurement system comprises a time synchronization controller (main synchronization controller), image acquisition synchronization controller, laser scanning synchronization controller, and external event recording synchronization controller. As shown in Fig. 7, DMI, GPS, and IMU generate corresponding distance pulses, second pulses, and other analogue signals, as well as digital signals like time. These signals are transmitted according to the requirements of each component in the data collection system. Since the mobile measurement system operates under dynamic conditions, to achieve higher time accuracy for data fusion and alignment, the sensor control signals are pulse electrical signals rather than data commands. Such signals can be directly introduced into the internal hardware circuits of the sensors. With a transmission speed akin to the speed of light, they enable “real-time” operation.

The information corresponding to each pulse, including the pulse occurrence time and serial number, is simultaneously transmitted to both the time synchronization controller and the POS (Positioning Orientation System) system. The POS system records in real time the data acquisition time stamp, as well as the position and attitude information of the vehicle platform in the geodetic coordinate system. The time synchronization controller is responsible for receiving the entire dataset and achieving system integration and synchronization control. It modulates and amplifies the distance pulses according to the preset pulse width and uses them as trigger signals to control the synchronous data acquisition by the CCD camera and the laser scanner. Simultaneously, these data are transmitted in real time to the data acquisition computer, facilitating the computer’s identification of the data transmitted back by the CCD camera and laser scanner, and for data storage. The main functions of the image acquisition synchronization controller, laser scanning synchronization controller, and external event recording synchronization controller are to receive synchronization information from the time synchronization controller and data acquisition parameters from the master computer. After integrating this information, they control and record the operational status of the sensors, and provide the sensors’ synchronization information to the data acquisition computer.

## Data accuracy verification

The software algorithm is another component of the detection device, primarily comprising a data acquisition system and a data processing system. The data acquisition system, installed on the control computer, connects to circuits, interfaces, memory units, and sensors. It is responsible for data collection, display of the collection status, and data storage. The memory of the storage unit is 8 GB, and the external storage capacity is 2 TB. Considering the large volume of point cloud and image data, as well as the limitations of the device’s size, the data processing part, functioning as a post-processing part, will complete its tasks in the laboratory after data collection. Its processing content is mainly defect detection algorithms, specifically includes multi-data fusion, convergence deformation calculation, and crack identification.

### Accuracy test for obtaining tunnel convergence disease information based on point cloud data

In order to verify that the point cloud data collected by the equipment in this paper can be used in the actual tunnel convergence disease detection work, this paper proposes a tunnel convergence calculation algorithm to process the point cloud data. In addition, since most of the tunnel convergence detections in the existing market use total station measurements, the convergence data extraction results in this paper are compared with the total station detection data.

**Fig. 8:**
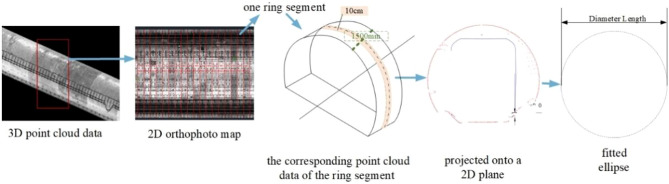
Convergence extraction process.

Tunnel convergence refers to the deformation value of a tunnel ring section around the center point, that is, the circumference of the ring is basically unchanged and the shape of the deformed ring section tends to be elliptical. In this paper, the point cloud data are used for curve fitting to obtain the fitted ellipse that best fits the shape of the deformed section. As shown in Fig. 8, the distance between the two sides of the ellipse center point is further calculated as the diameter length of the ellipse.

The deformation values of ring sections are calculated one by one. Specifically, a 2D orthophoto map of the tunnel surface is obtained by projecting the three-dimensional point cloud data onto a two-dimensional plane. Then, a ring segment can be found between two splice seams in an orthophoto image. Finally, the corresponding point cloud data of the ring segment is extracted according to the projection relationship between the orthophoto image and the point cloud.

Since the width of the ring segment is 1500 mm, the point cloud data of a ring is large, which increases the computational workload. Therefore, in this paper, the center part of each ring segment is taken for calculation. We take 10 cm wide point cloud data around the center point of the ring, which is projected onto a 2D plane and denoised by wavelet filtering.

As shown in formulae (3−1) and (3−2)^26^, there are two variables in the wavelet transform: the choice of scale α determines the spatial size range of the analyzed tunnel contour features. Small scale α focuses on subtle local deformations, while large-scale α reflects overall contour deviation patterns at the meter level or even longer. The translation amount τ controls the position of the wavelet function moving along the tunnel axis. When the scale and position of the wavelet function highly match the specific spatial geometric features of the tunnel contour at that position, the wavelet coefficients will reach a local maximum. This indicates that the irregularity of the contour of that specific type and size exists at that particular axis position. By calculating the wavelet coefficients of all scales α and positions τ, we obtained the spatial frequency distribution map of the tunnel. This distribution clearly reveals the distribution of different spatial dimensions from subtle defects to overall deformation along the tunnel axis, thereby achieving effective noise filtering and effective extraction of target features.

3-1 $$\:F\left(\omega\:\right)={\int\:}_{-\infty\:}^{+\infty\:}f\left(t\right)\times\:{e}^{-i\omega\:t}dt$$

3-2 $$\:\text{W}\text{T}({\upalpha\:},{\uptau\:})=\frac{1}{\sqrt{{\upalpha\:}}}{\int\:}_{-{\infty\:}}^{+{\infty\:}}\text{f}\left(\text{t}\right)\times\:{\Phi\:}\left(\frac{t-{\uptau\:}}{{\upalpha\:}}\right)d\text{t}$$

The denoised cross-section point cloud is curve-fitted using the least squares method^22–25^ to obtain a fitted ellipse that best fits the shape of the cross section. Then long axis of an ellipse is calculated according to the formula (3–3)–(3–5)^26^.

3-3 $$\:A{x}^{2}+Bxy+C{y}^{2}+Dx+Ey+1=0$$

3-4 $$\:{X}_{c}=\frac{BE-2CD}{4AC-{B}^{2}},\:{Y}_{c}=\frac{BD-2AE}{4AC-{B}^{2}}$$

3-5 $$\:{a}^{2}=\frac{2\left(A{x}_{c}^{2}+C{Y}_{c}^{2}+B{X}_{c}{Y}_{c}-1\right)}{A+C+\sqrt{{\left(A-C\right)}^{2}+{B}^{2}}}$$

where ($\:{X}_{c}$, $\:{Y}_{c}$) are the coordinates of the geometric center of the ellipse and $\:a$ is the long axis of the ellipse, also the distances in the left and right horizontal directions from the center point of the ellipse.

Then, the distance from each point to the fitted ellipse is calculated and the points are selected based on a threshold value (> 0.01 m), thus removing the noisy point cloud from the section point cloud. The remaining points go through an iterative process of ellipse fitting and distance exclusion several times until no point is excluded, and the final ellipse fitted is the tunnel cross-section. The above process is implemented using Python.

### Accuracy test for obtaining information of crack damage on tunnel surface based on image data

As one of the main diseases in tunnels, most cracks present as fine and thin lines. Since the low accuracy of crack identification using laser point cloud does not meet the need of regular inspection, Most of the existing literature uses image recognition to extract crack information. In order to show that the accuracy of the images captured by the equipment in this paper can meet the requirements of crack detection, the crack widths in the pictures are extracted and compared with the actual measurements.

The core algorithm for crack recognition in this study is based on image processing techniques. In general, image-based crack detection methods can be divided into three categories: traditional digital image processing, machine learning, and deep learning approaches. Traditional methods often involve steps such as noise reduction, edge detection, thresholding, and morphological operations. While computationally efficient, these methods usually require hand-crafted features and are sensitive to lighting variations and noise. Machine learning-based methods employ classifiers such as SVM or random forests, but still depend on manually designed features. In contrast, deep learning models, especially convolutional neural networks (CNNs), can automatically learn discriminative features from raw pixel data and have demonstrated superior performance in accuracy and robustness under complex conditions. Therefore, considering the fine and irregular nature of tunnel cracks, this paper adopts the Mask R-CNN architecture, an instance segmentation deep learning model, to achieve precise crack detection and segmentation.

**Fig. 9:**
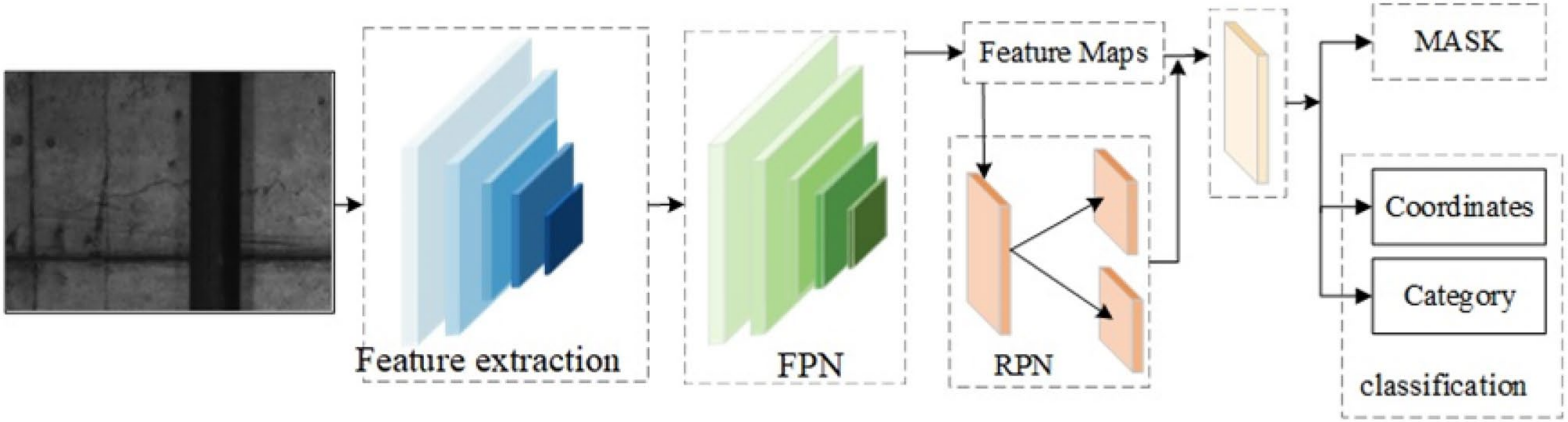
Crack extraction process.

The MASK RCNN deep learning network is used for training, and the training and testing sets are divided into a 7:3 ratio. MASK R-CNN consists of the following parts: Feature Extraction, Feature Pyramid Network (FPN), Region Proposal Network (RPN), Regression Classification Branch, and Masking Branch. The data processing flow is shown in Fig. 9.

For feature extraction, we employed a Residual Neural Network (ResNet101) as the backbone. The architecture incorporates skip connections to mitigate the degradation problem that often occurs as network depth increases, thereby stabilizing training and enhancing feature propagation. Multi-scale feature extraction was achieved using convolutional kernels of sizes 7 × 7, 14 × 14, 28 × 28, 56 × 56, and 112 × 112, allowing the model to capture both coarse and fine crack characteristics. These features were further refined and integrated through a Feature Pyramid Network (FPN), which enhances detection across different scales by combining high-resolution shallow features with semantically rich deep features.

Region proposals were generated using a Region Proposal Network (RPN), which scans over the feature maps to identify candidate regions containing potential cracks. Each proposal was then processed through ROI Align to extract fixed-size feature maps (7 × 7 pixels), preserving spatial accuracy and avoiding misalignment issues inherent in earlier methods like ROI Pooling.

The network branches into two output pathways: a classification and regression branch, and a mask prediction branch. The classification branch employs a SoftMax classifier to categorize regions as either crack or background, simultaneously performing bounding box regression to refine the coordinates of each detected crack. The second branch utilizes a fully convolutional network to predict pixel-accurate binary masks, enabling precise segmentation of crack morphology.

**Fig. 10:**
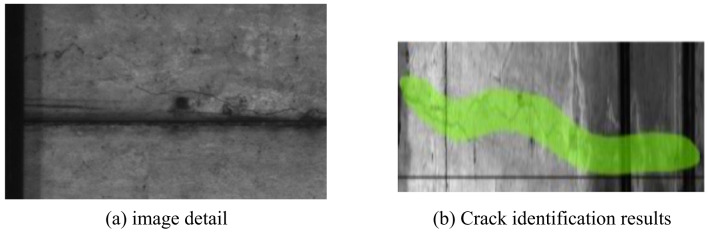
Fracture identification.

To avoid the problem of poor recognition performance caused by interference from other linear interferences in the tunnel, we annotated other current targets that are prone to recognition errors while annotating cracks, as shown in Fig. 10, to obtain real crack textures, interference noise textures, and crack background information. This not only increases the computational cost of the network, but also avoids false detection as much as possible.

**Fig. 11:**
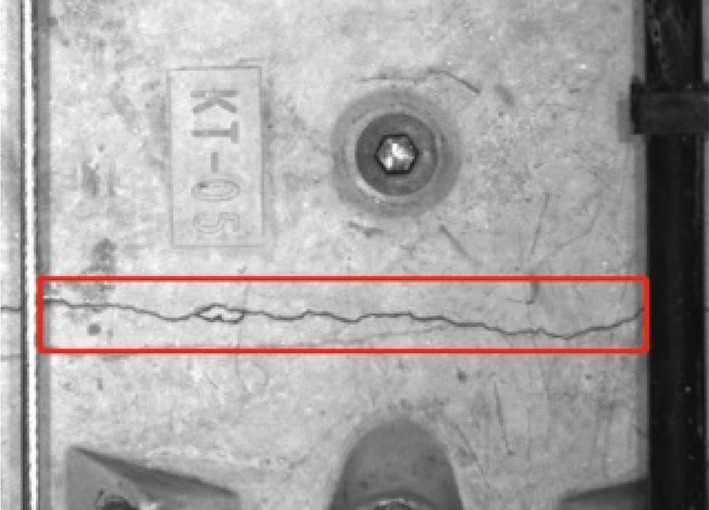
Samples of crack.

Crack width extraction is then performed, we randomly select the cracks in the image as shown in Fig. 11. The number of pixels occupied in the width direction of each crack is counted.

**Fig. 12:**
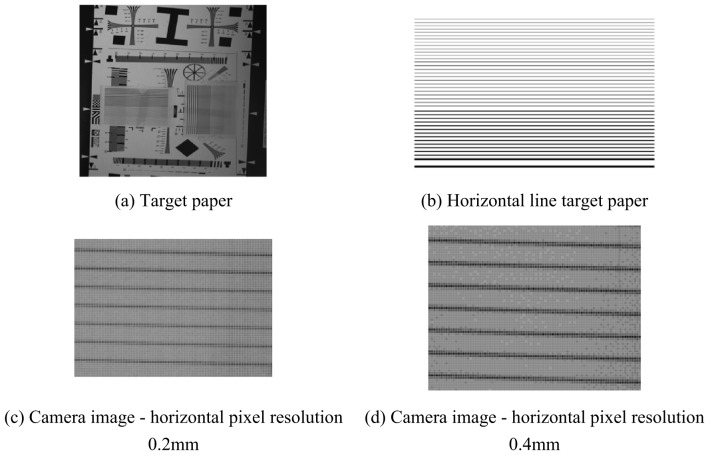
Target paper and camera image.

Next, the actual width represented by a pixel in the image needs to be calculated, taking camera 1 as an example. The image of the target paper in the proposed application environment is captured, as shown in Fig. 12a, and the target paper is printed on A4 paper with the width of the horizontal lines of 0.2 mm, 0.4 mm, 0.6 mm, and 1 mm, as shown in Fig. 12b.The cameras are placed horizontally on the table, the target paper is placed vertically in front of the camera, and the distance to the target paper is adjusted using a laser rangefinder to simulate the positional relationship in the actual operating environment. The camera shoots the target paper respectively and enlarges the camera’s horizontal pixel to 0.2 mm and 0.4 mm. for example, the imaging effect is shown in Fig. 12c,d. According to the measured site light environment and other factors to consider, the actual processing of an obvious pixel crack width of 0.2 mm. It is possible to find the minimum value of the pixel in the direction of the width, so it is known that the width of an obvious pixel crack is 0.4 mm. the multiplication of the pixel width with the number of pixels is sufficient to calculate the value of the width of the crack.

## Registration algorithm for point clouds and images

As smart cities and digital construction evolve, the construction of 3D visualization models is garnering increasing attention. Point clouds provide geometric structural information of objects, while images offer texture and color information. The registration of these two enhances the comprehensive recognition of objects. This paper introduces a method where, following the 3D reconstruction of terrestrial models from laser scanning data, the color images obtained by CCD cameras are automatically mapped onto the 3D models in a one-to-one correspondence, thus creating realistic 3D models. A key issue in this process is the spatiotemporal registration of CCD images with laser scanning data.

### Time registration

Due to the differing frequencies at which the various sensors in a mobile 3D measurement system operate, each device maintains its own independent time system. For instance, the collection frequency of laser scanning data is extremely high, ranging from 10 to 100 kHz, and it samples at equal time intervals. In contrast, the CCD camera captures images under the control of a synchronizer at equal spatial intervals, with a collection frequency of only a few Hertz. Consequently, it is not possible to match the two on a time axis. The purpose of time registration is to establish and maintain a unified time baseline, ensuring that each laser point and each image can obtain real-time coordinates and attitude data of the moving carrier at the time stamp of data generation.

**Fig. 13:**
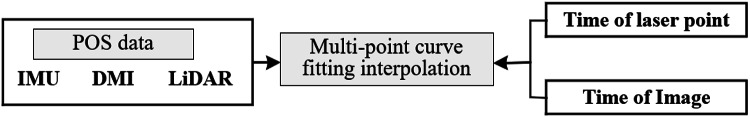
Temporal registration algorithm.

The output frequency of the IMU is 200 Hz, the output frequency of the camera is typically once every few seconds, and that of the laser scanner ranges from 10 kHz to 1 MHz. The temporal accuracy of both these systems is lower than that of the POS system’s output. Consequently, by using the moving vehicle’s position and attitude information from the POS output as a basis, and interpolating the timestamps of each point in the laser scanner point cloud and the capture times of camera images within the POS data, it is possible to obtain the position and attitude information of the vehicle corresponding to each laser point and each digital image, as illustrated in the Fig. 13.

When the POS system outputs the carrier’s pose information at a frequency of 200 Hz, it can be considered that the carrier moves in a uniform linear motion within the interval between two outputs (0.005 s). Therefore, in the process of temporal registration, the interpolated values can be directly obtained by using a multi-point curve fitting interpolation method.

### Spatial registration

Although multiple sensors are mounted on the same platform for data collection, the coordinate systems of the data they acquire are different. For instance, the GNSS receiver uses the WGS84 coordinate system for its data, the IMU utilizes an inertial coordinate system centered on itself, and the laser scanner adopts a scanning coordinate system with the laser emission center as its origin. Cameras with internal orientation elements use a photographic coordinate system centered on the lens focus. To unify the target’s three-dimensional information in a same coordinate system, it is necessary to standardize the spatial reference and perform spatial alignment.

**Fig. 14:**
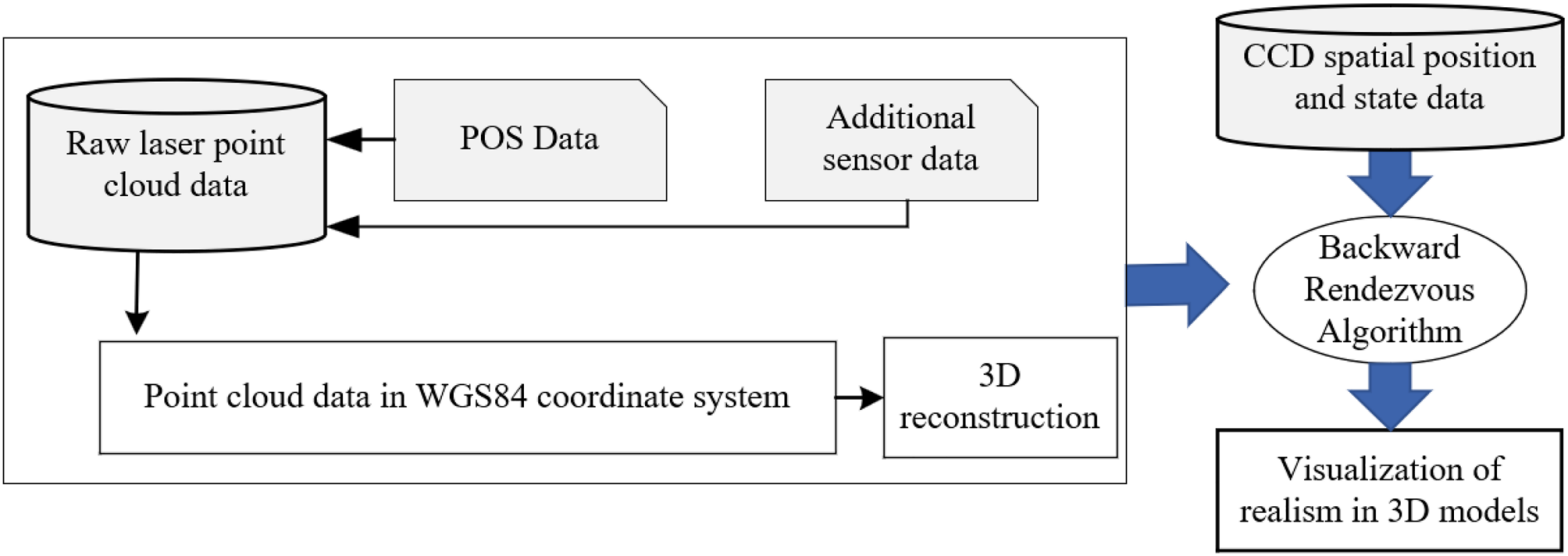
Spatial registration method.

Firstly, during the fusion process of mobile 3D laser scanning data with sensor placement parameters and POS data, the coordinates of ground points are sequentially transformed into the reference coordinate systems of laser scanning, inertial platform, local horizontal, and local vertical, and finally converted into the WGS-84 coordinate system, forming spatial point cloud data. Then, as shown in the Fig. 14, these data are then feature extracted to identify key points and carried out 3D reconstruction. This results in the creation of three-dimensional models of terrestrial object. After completing the 3D reconstruction of terrestrial objects, the WGS84 coordinates of their features or key points can be obtained. Furthermore, based on the spatial position and orientation of the CCD camera at the time of capturing the target, the pixel coordinates of the point on the CCD image are determined through the single camera’s backward resection algorithm, thereby obtaining the color information of the point.

**Fig. 15:**
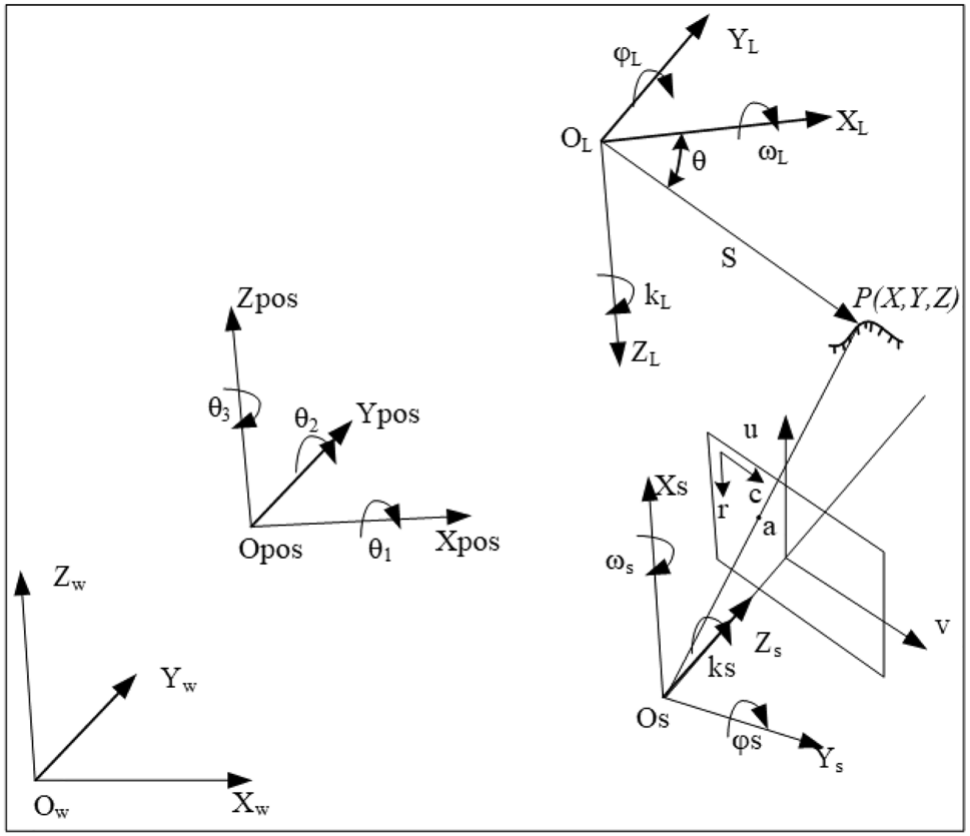
The relationships between four coordinate systems.

As shown in Fig. 15, ($\:{\text{O}}_{\text{P}\text{O}\text{S}}^{}-{X}_{POS}^{}{Y}_{POS}^{}{Z}_{POS}^{}$) represents the POS coordinate system, ($\:{\text{O}}_{\text{S}}^{}-{X}_{S}^{}{Y}_{S}^{}{Z}_{S}^{}$) indicates the CCD camera imaging coordinate system, ($\:{O}_{L}^{}-{X}_{L}^{}{Y}_{L}^{}{Z}_{L}^{}$) denotes the laser scanning coordinate system, and ($\:{O}_{w}^{}-{X}_{w}^{}{Y}_{w}^{}{Z}_{w}^{}$) stands for the WGS84 coordinate system.

The time stamp of the laser scanning point P is denoted by $\:{t}_{l}$, and the coordinates of P in the WGS84 derived from the laser scanning point can be expressed as Eq. (4−1):

4-1 $$\:\left[\begin{array}{c}{X}_{w}^{l}\\\:{Y}_{w}^{l}\\\:{Z}_{w}^{l}\end{array}\right]=\left[\begin{array}{c}{X}_{POS}^{w-{t}_{l}}\\\:{Y}_{POS}^{w-{t}_{l}}\\\:{Z}_{POS}^{w-{t}_{l}}\end{array}\right]+\left[\left[\begin{array}{c}{X}_{l}^{POS}\\\:{Y}_{l}^{POS}\\\:{Z}_{l}^{POS}\end{array}\right]+{R}_{l}^{POS}\left[\begin{array}{c}{d}_{l}\text{sin}{\theta\:}_{l}\\\:{d}_{l}\text{cos}{\theta\:}_{l}\\\:0\end{array}\right]\right]$$

[$\:{X}_{POS}^{w-{\text{t}}_{\text{l}}}$, $\:{Y}_{POS}^{w-{\text{t}}_{\text{l}}}$, $\:{Z}_{POS}^{w-{\text{t}}_{\text{l}}}$]T is the coordinate vector of point P in the POS system, [$\:{X}_{l}^{POS}$, $\:{Y}_{l}^{POS}$, $\:{Z}_{l}^{POS}$]T is the coordinate vector of the origin of the POS coordinate system in the laser coordinate system, and $\:{R}_{l}^{POS}$ is the rotation matrix for transforming the point cloud from the POS coordinate to the laser coordinate. Suppose the time stamp at which the CCD camera captures point P is denoted as $\:{t}_{s}$, and the coordinate of P in the camera coordinate system is ($\:{X}_{s}$, $\:{Y}_{s}$, $\:{Z}_{s}$), then the coordinate of P in the WGS84 coordinate system can be calculated by Eq. (4−2):

4-2 $$\:\left[\begin{array}{c}{X}_{w}^{s}\\\:{Y}_{w}^{s}\\\:{Z}_{w}^{s}\end{array}\right]=\left[\begin{array}{c}{X}_{POS}^{w-{t}_{s}}\\\:{Y}_{POS}^{w-{t}_{s}}\\\:{Z}_{POS}^{w-{t}_{s}}\end{array}\right]+\left[\left[\begin{array}{c}{X}_{s}^{POS}\\\:{Y}_{s}^{POS}\\\:{Z}_{s}^{POS}\end{array}\right]+{R}_{s}^{POS}\left[\begin{array}{c}{X}_{s}\\\:{Y}_{s}\\\:{Z}_{s}\end{array}\right]\right]$$

[$\:{X}_{POS}^{w-ts}$, $\:{Y}_{POS}^{w-ts}$, $\:{Z}_{POS}^{w-ts}$]T is the coordinate vector of P in the POS system, and [$\:{X}_{s}^{POS}$, $\:{Y}_{s}^{POS}$, $\:{Z}_{s}^{POS}$]T is the coordinate vector of the camera coordinate system’s origin in the POS coordinate system. $\:{R}_{s}^{POS}$ is the rotation matrix of the point transformed from the POS coordinate system to the camera coordinate. Utilizing Eq. (4−3), the pixel coordinates of the point on CCD image can be determined:

4-3 $$\:\left[\begin{array}{c}{X}_{s}\\\:{Y}_{s}\\\:{Z}_{s}\end{array}\right]={\left[{R}_{s}^{POS}\right]}^{-1}\left[{\left[{R}_{s}^{POS}\right]}^{-1}\left[\left[\begin{array}{c}{X}_{POS}^{w-{\text{t}}_{\text{l}}}\\\:{Y}_{POS}^{w-{\text{t}}_{\text{l}}}\\\:{Z}_{POS}^{w-{\text{t}}_{\text{l}}}\end{array}\right]+{R}_{s}^{POS}\left[\left[\begin{array}{c}{X}_{l}^{POS}\\\:{Y}_{l}^{POS}\\\:{Z}_{l}^{POS}\end{array}\right]+{R}_{s}^{POS}\left[\begin{array}{c}{d}_{l}\text{sin}{\theta\:}_{l}\\\:{d}_{l}\text{cos}{\theta\:}_{l}\\\:0\end{array}\right]\right]-\left[\begin{array}{c}{X}_{POS}^{w-{\text{t}}_{\text{s}}}\\\:{Y}_{POS}^{w-{\text{t}}_{\text{s}}}\\\:{Z}_{POS}^{w-{\text{t}}_{\text{s}}}\end{array}\right]\right]-\left[\begin{array}{c}{X}_{s}^{POS}\\\:{Y}_{s}^{POS}\\\:{Z}_{s}^{POS}\end{array}\right]\right]$$

Based on the imaging formula, it can be concluded as Eq. (4–4):

4-4 $$\:\left\{\begin{array}{c}{r}_{p}=\frac{{X}_{s}}{{Z}_{x}}\times\:{f}_{u}+{r}_{0}\\\:{c}_{p}=\frac{{Y}_{s}}{{Z}_{x}}\times\:{f}_{v}+{c}_{0}\end{array}\right.$$

where $\:{r}_{p}$ and $\:{c}_{p}$ are the horizontal and vertical coordinates of the imaging point a, u is the object distance, v is the image distance, and f is the focus length. For a region formed by several spatial points on the model, it can also be mapped onto a polygonal area on the CCD image. By using interpolation methods, the texture color information can be mapped onto the 3D model, thereby achieving realistic visualization of the 3D mode.

## Experiment and results

To ensure that the equipment could perform the expected inspection work, we tested it in a real metro shield tunnel in Wuhan, China. Each tunnel ring has a design internal diameter length of 5.5 m and a design external diameter of 6.2 m.

### On-site metro tunnel inspection

With reference to existing research articles^8,9^, we consider two important metrics when evaluating the performance of equipment: efficiency and accuracy. Efficiency refers to the time spent debugging the system and the time spent assembling and disassembling the equipment. Accuracy refers to the difference between the real defects and the disease information extracted by the equipment and methods proposed in this paper. Since the deformation of the tunnel has occurred during operation and its true value is difficult to know, and the total station deformation monitoring method has a reliable accuracy and is still widely used in practical projects^27,28^, we choose the total station method to measure the diameter of the tunnel and compare its measurement results with those obtained in this paper.

**Fig. 16:**
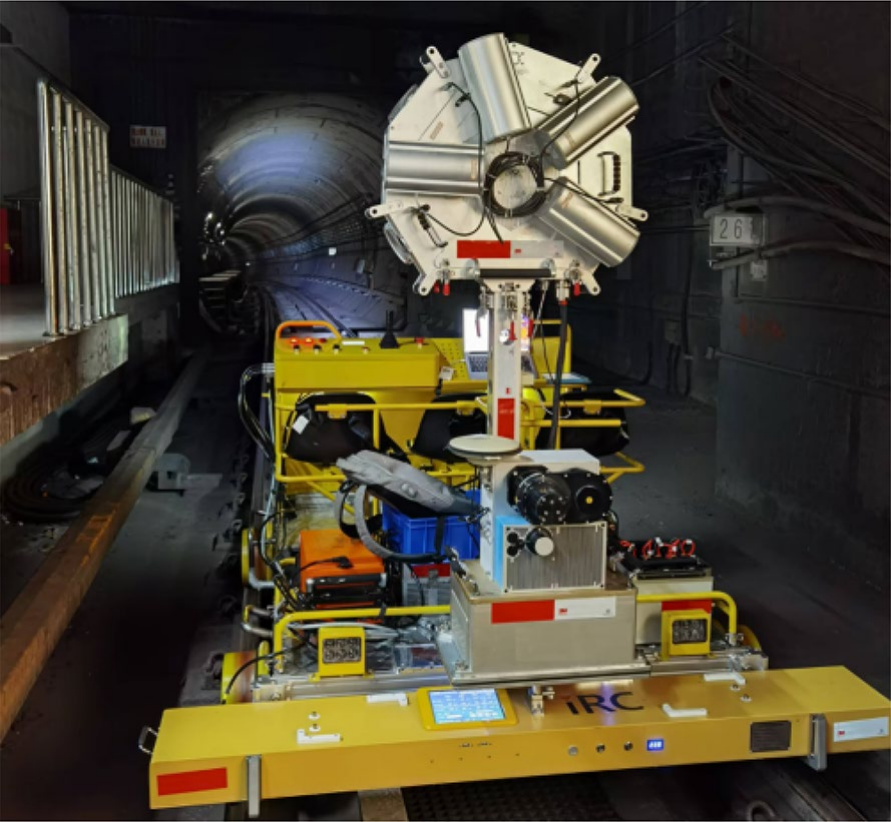
On-site detection.

In terms of efficiency, four inspectors used the equipment to complete tunnel inspections, as shown in Fig. 16, acquiring more than 100 G of laser point cloud data and high-resolution images from 10 stations and 9 intervals (about 20 km) in about 2.5 h. It takes 25 min to set up the equipment and make it operational. And the time to disassemble the equipment after inspection is about 10 min. In order to obtain highly accurate data, the speed of the equipment was approximately 15 km/h on the straight section and 5 km/h on the curved section. The total time was 2.5 h.

About accuracy. We choose one of the intervals in the inspected metro line for data processing to extract the disease information. The interval has a total of 559 loops and a length of about 0.8 km.

#### Results of convergence deformation calculations

**Fig. 17:**
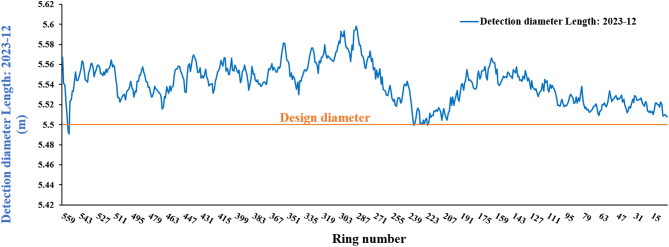
Diameter of the tunnel ring piece.

Using the point cloud data and the method described in Sect. 3.2 to calculate the tunnel deformation. Then the long axis values of each ring piece are extracted as shown by the blue line in the Fig. 17.

The total station automatic deformation monitoring method^25^ has a reliable accuracy and is still widely used in practical projects. The current tunnel convergence detection work is mainly carried out using a total station. Therefore, in order to verify the accuracy of the data, the detection results of the equipment proposed in this paper are compared with those of the Leica TM50 total station. The equipment and software used in the comparison experiments include Leica TM50 total station and its own tunnel section measurement software. In any surface mode, a range accuracy of 2 mm + 2ppm can be achieved. Leica Infinity 3.6.1r software was selected for internal processing of the tunnel section data. The test tunnel is a tunnel with a large gradient and a small turning radius between two stations of Hangzhou Metro Line 1, and part of the section in this interval has a large deformation, which is representative. Starting from the 10th ring of the tunnel, a section is measured every 10 rings. The operation steps of total station measurement are as follows:

A total station is first set up on the center line of the first section to be measured. The section to be measured is selected in the center of each ring of pipe sections, avoiding obstructions as far as possible. The prism-free mode is set and the known point rear view orientation is used.

(2) Determine the position of the section. In order to accurately measure the diameter of the tunnel section, it is necessary to first determine a measurement datum plane perpendicular to the tunnel. The specific methods for determining the reference plane are as follows:

① Firstly, use the laser scanner to locate the position of the ring piece. According to the laser in the current ring on the left and right sides of the laser line on both sides of the wall on either side of a target paper, spray paint marking points on the lower track surface to determine the position of the total station set up.
② Set up the total station on the color marking point.

Total station alignment levelling, laser point aligned with the target paper, and make the eyepiece in the approximate horizontal direction, the use of continuous ranging function to fine-tune the horizontal angle, to find out the shortest distance will be measured when the horizontal angle is set to zero.

③ At this time, spray marking on the laser point, consider it as the rear view point, and then keep the horizontal angle unchanged to adjust the vertical angle of the total station to the other side of the tube sheet near the symmetrical position, and make a good marking as a control point. Then, keeping the horizontal angle unchanged, adjust the vertical angle of the total station to a nearly symmetrical position on the other side of the tube sheet and make a good mark as a check point.

(3) Section measurement parameter setting.

On the tunnel section measurement software, three points (start position, termination position and zenith position) are set. In the measurement parameter setting interface, you need to enter the section measurement step, usually set to 15 ~ 20 cm, and also need to enter the ellipse radius limit value, usually set to 1 m, the limit value is set too small, it is easy to filter out the valid points.

The comparison results are shown in the Table 1.

**Table 1:** Comparison results.

Comparative metrics		The values of subtraction (s)		
		s < 2 mm	2 mm ≤ s < 3 mm	3 mm < s ≤ 4 mm
Horizontal diameter	Number of rings	25	13	2
	Percentage of total rings	62.5%	32.5%	5.0%
Maximum diameter	Number of rings	27	11	2
	Percentage of total rings	67.5%	27.5%	5.0%

The difference in disease extraction results between the method described in this paper and the total station measurement results is within 2 mm in over 60% of cases, with differences less than 3 mm in 95% of cases. In addition, we found in our statistics that the total station measurement results are generally larger than the 3D laser scanning results in the whole line, mainly because when the total station measures the section, the scanning section cannot be completely perpendicular to the axis of the tunnel, resulting in large scanning results, and especially the deviation of the total station measurement results at the tunnel bends is a bit more obvious. In this comparison, the sections with subtraction values of more than 3 mm are all located in the tunnel curves (with the radius of the curve *R* = 400 m), while in the straight-line section, the coincidence is better. All in all, it can be concluded that the 3D laser scanning results are reliable.

#### Camera accuracy test results - crack width

**Fig. 18:**
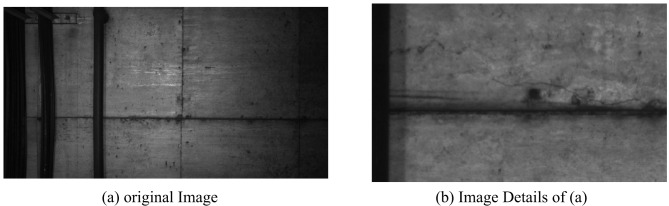
Images acquired through the CKY-200.

The detection standard of cracks requires that we need to detect cracks with a width of at least 0.2 mm. As shown in the Fig. 18, the resolution of the captured image is 4096px×2048 px, due to the lack of light in the metro tunnel, the overall image tone is dark, but the information of cracks, leakage and falling blocks can still be clearly seen from the captured image.

Five cracks were randomly selected and the number of 20-width pixels per crack was counted. The maximum width and the average value of the width for each crack were calculated and compared with the actual values. The results are presented in Table 2.$\:{Max}_{\left(c\right)}$ denotes the maximum width of the crack, $\:{Max}_{\left(true\right)}$denotes the actual width of the crack, $\:{AVG}_{\left(c\right)}$ denotes the average width of the crack, $\:{AVG}_{\left(true\right)}$ denotes the actual average width of the crack. The results demonstrate that the maximum discrepancy between the calculated and actual maximum width values is 0.093 mm, while the average discrepancy is 0.104 mm. The overall error accuracy is within 5%.

**Table 2:** A statistical comparison of maximum and average values of crack width measurements with actual values.

Standard	Crack number				
	①	②	③	④	⑤
$\:{Max}_{\left(c\right)}$	2.15	2.15	1.72	2.15	2.58
$\:{Max}_{\left(true\right)}$	2.193	2.21	1.813	2.214	2.496
$\:{AVG}_{\left(c\right)}$	1.92	1.78	1.505	1.89	2.104
$\:{AVG}_{\left(true\right)}$	2.012	1.738	1.563	1.833	2.17

Overall, the equipment can quickly scan the entire line, collect large amounts of high-precision and comprehensive multi-type data, and provide various ways of presenting tunnel information. And the use of mobile inspection equipment for data acquisition can greatly reduce labor and improve inspection efficiency compared to traditional methods. In addition, regular scanning of tunnels over a long period of time can also provide a massive database for analyzing the long-term performance of tunnels.

#### Registration of image and point cloud

**Fig. 19:**
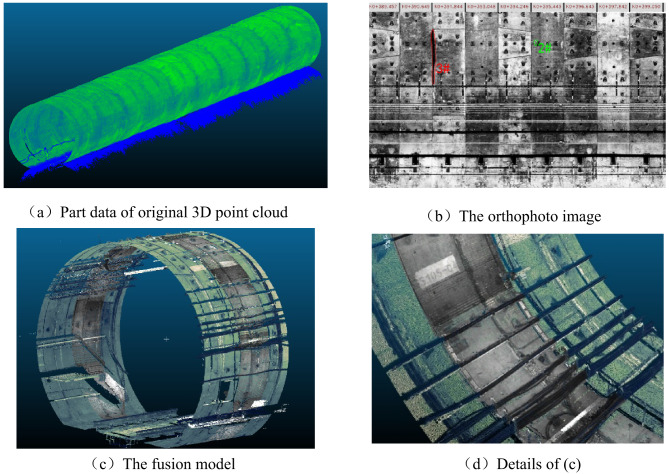
Point cloud 3D reconstruction data display.

Figure 19a is part of original point cloud data, which has an absolute accuracy of less than 20 mm^29^. Figure 19b shows the part of the orthophoto image, a projected two-dimensional plane image of Fig. 19a, which is displayed by assigning different colors according to the depth value of point cloud. And a single pixel point representing the actual tunnel area of about 2 mm×2 mm. The point cloud data can completely rebuild the real tunnel lining surface. And the tunnel structures are clearly visible, with no distortions or strain deformations, and details such as suspected water leaks or falling blocks can be identified (marked in Fig. 19b 2# and 3#). Figure 19c shows the fusion of a high-resolution image and the corresponding point cloud data according to the method described in Sect. 3.1, and Fig. 19d shows the enlarged details of Fig. 19c. Compared to Fig. 19a, the 3D tunnel model with added image information has a stronger 3D visual effect and realism, making better use of the respective strengths of images and point clouds, and can show more detailed information. As shown in Fig. 19d, the texture and the numbers of the lining surface can be shown more clearly.

It is important to note that the registration method proposed in this paper is intended as a 3D real modelling reference and is not designed for use in the accurate reconstruction of 3D tunnels.

**Fig. 20:**
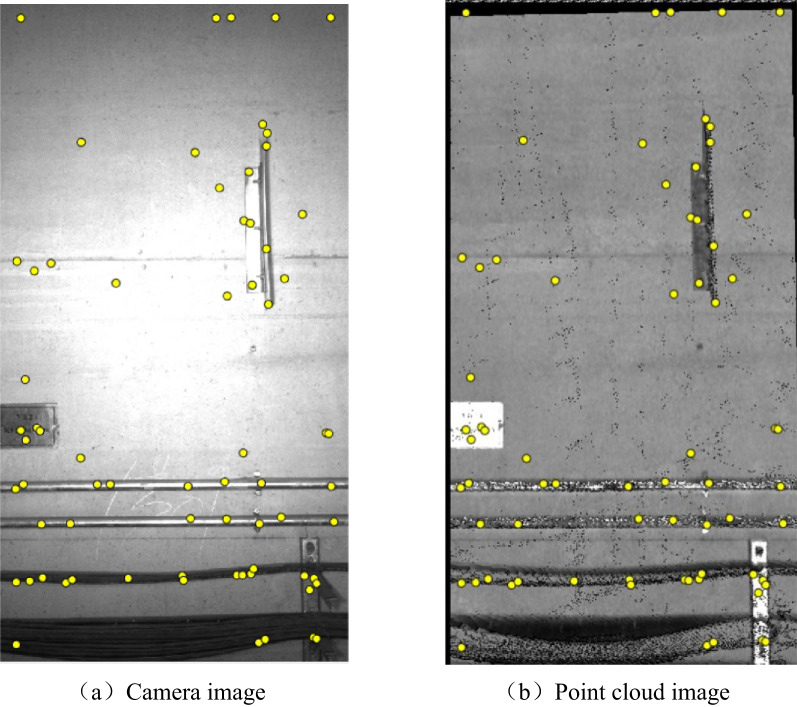
Homologous image points after alignment.

Multiple homonymous image points are searched in the aligned Point cloud image and Camera image as shown in Fig. 20 and the corresponding pixel values of the homonymous points in the image are calculated, as the size of each pixel point is 2 mm × 2 mm, thus the error after alignment is calculated according to the difference of the pixel points. As shown in Table 3 and 690 homonymous points were selected and the corresponding pixel coordinates in Point cloud image and Camera image were calculated respectively, thus calculating the distance difference between the two pixel points. According to Table 3, the average error of the calibration is 3.3146 mm.

**Table 3:** The distance difference between the two pixel points.

Camera image coordinates X	Camera image coordinates Y	Point cloud image coordinates X	Point cloud image coordinates Y	Distance
351	803	353	803	2.0000
403	810	404	810	1.0000
406	746	407	747	1.4142
438	800	438	800	0.0000
516	751	516	752	1.0000
520	808	520	807	1.0000
26	899	26	901	2.0000
48	898	47	899	1.4142
67	893	67	894	1.0000
103	902	103	902	0.0000
113	897	113	897	0.0000
199	896	200	895	1.4142
284	894	284	891	3.0000
286	902	286	898	4.0000
369	894	369	890	4.0000
…	…	…	…	…
375	895	378	890	5.8310
			Average distance	3.3146

### Discussion

#### Point cloud accuracy validation

In order to provide further evidence that the device proposed in this paper is capable of acquiring higher accuracy point cloud data, the accuracy of the scanner is verified in this paper using a volume block static measurement method.

**Fig. 21:**
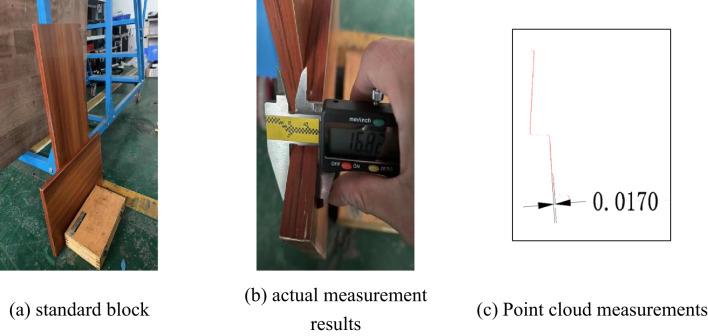
Measurement results.

Firstly, the standard measuring block was measured and its true width was determined to be 16.82 mm, as illustrated in Fig. 21. Subsequently, the block was scanned with the aforementioned equipment at 1 m, and the resulting data was converted into a single-frame section point cloud. This section data was then imported into CAD, where the thickness of the standard block was measured to be 17 mm.

Then take a piece of standard block and measure the thickness of the block using digital vernier calipers, the three measurements are 20.71 mm, 20.62 mm, 20.66 mm and the average value is 20.66 mm.

Place the standard block at a position approximately 2.5 m away from the equipment and parallel to the direction of the equipment’s movement. The device scans the point cloud data of the standard block’s position and processes it, outputting the average thickness of the measured edge of the standard block. Compare the output value with the measurement value of the digital vernier caliper, calculate the error value of the point cloud measurement accuracy, as shown in Table 4, with a maximum error of 0.39 mm.

**Table 4:** Comparison of measurement data.

Measurement number	Standard thickness (mm)	Measured thickness (mm)	Error value (mm)
1	20.66	20.71	0.05
2		20.49	-0.17
3		20.27	-0.39
4		20.39	-0.27
5		20.62	-0.04

#### Comparison of equipment with existing products of the same type

Comparing the CKY-200 and existing similar devices in terms of speed, accuracy and variety of data collection. As shown in the Table 5, some of the existing devices are still manually operated and equipped with only a single type of sensor, such as the GRP 5000^30^ and Leica SiTrack^31^, equipped with a laser scanner, which can be used to identify leakage, falling blocks and deformation of the tunnel, but its accuracy does not meet the requirement of crack identification. Equipment capable of acquiring point cloud and image data simultaneously includes MIMM-R^32^, Spacetec: TS 4^33^, Tunnelings^34^. However, these devices are too bulky and expensive and therefore not suitable for underground inspections. The CKY-200 is equipped with 8 sets of CCD cameras and a laser scanner, the detection range of the sensors is 290° and 360° respectively, which can cover the inspection part of the tunnel. And the equipment uses a vehicle mobile mode, which can be driven autonomously on the track. Considering the detection accuracy of small cracks, the speed of the equipment is limited to 15 km/h on the straight and 5 km/h on the corner, which is still a high inspection speed. In addition, the equipment is designed to be detachable, allowing for quick assembly and disassembly.

**Table 5:** Comparison between typical equipment.

Device	Moving vehicle	Core sensors	Maximum speed	Number of cameras	Cracks detection	Equipment display
CKY-200	√	CCD, laser	15 km/h on the straight section and 5 km/h on the curved section	8	0.2 mm	
GRP5000	×	Laser	0.7 km/h	-	0.3 mm	
MIMM-R	√	CCD, laser, GPR	50 km/h	8	0.3 mm	
ROBOSPECT	√	CCD	Discontinuous inspection	2	0.5 mm	
Spacetec: TS4	√	CCD, laser	5 km/h	-	0.3 mm	
Tunnelings	√	CCD, laser	30 km/h	6	Unknown (images of 1024 × 4096 pixels)	
Leica SiTrack	×	Laser	6.4 m/s	-	0.31 mm	
MTI-200a^35^	×	CCD	10 km/h	6	0.29 mm	

## Conclusion

This paper designed the multi-sensor mobile inspection equipment and proposed the data processing algorithms. Through on-site experiments and comparisons, the accuracy, stability, and efficiency of the device have been verified, making it fully applicable for practical tunnel operation and maintenance, which can be concluded as follows:

The equipment integrates eight high-resolution CCD cameras and a high-precision laser scanner, which can cover the entire inspection area of the tunnel and solve the key problem of acquiring multiple types of data simultaneously. The main parameters of the device were compared with those of existing similar devices, and the results show that the CKY-200 is superior in terms of speed of movement, type of data acquired and high accuracy.

The device contains various sensors such as IMU and DMI, and can also obtain absolute attitude information on curved lines. It is less affected by curved lines in the extraction of data such as convergence and deformation. However, the lateral acceleration generated during curved sections affects the measurement accuracy of IMU, requiring a more complex dynamic compensation model. The sliding caused by the contact of the wheel flange on the curved section results in distortion of the odometer pulse count, and precise compensation is required for the difference in travel between the inner and outer rails. This paper proposes a 3D modelling method based on point cloud and image data, solving the key problem of how to align CCD images temporally and spatially with laser scanning data. Compared with the raw point cloud data, the 3D tunnel model with added image information has a stronger 3D visual effect and realism, and can show more detailed information. We hope that the constructed 3D visualization model can contribute to digital city construction.

Based on the data obtained by CKY-200, this paper proposes a deformation calculation method based on laser data and a crack width calculation method based on CCD images to verify that the accuracy of the data can meet the actual work requirements. The deformation test results of 40 sections are compared with the measurement results of the Leica TM50 total station, and the results show that 95% of the subtraction values between the two is less than 3 mm. The sections with subtraction values of more than 3 mm are all in the curved part of the tunnel, while the straight part has better agreement. This is due to the fact that the scanning sections of the total station in the curved part of the tunnel cannot be completely perpendicular to the tunnel axis, resulting in an obvious scanning deviation. In addition, the widths of several cracks identified in the images are measured in the actual tunnel, showing that the images can identify cracks as small as 0.2 mm.

## Data Availability

The datasets of this study are available from the corresponding author on reasonable request.

## References

[1] Gao, X. et al. Faster multi-defect detection system in shield tunnel using combination of FCN and faster RCNN. *Adv. Struct. Eng.* **22** (13), 2907–2921 (2019).

[2] Xu, Z. *Data Processing of Tunnel Deformation Monitoring* (Tongji University, 2009).

[3] WANG Shi-lei, G. A. O. et al. Review on inspection technology of railway operation tunnels. *J. Traffic Transp. Eng.* **20** (5), 41–57 (2020).

[4] Qing, A., Yong, Y. & Bi, X. Acquiring sectional profile of metro tunnels using charge-coupled device cameras. *Struct. Infrastruct. Eng.* **12** (9), 1065–1075 (2016).

[5] Gan, C. & Lei, Y. Tunnel deformation monitoring based on laser distance measuring and vision assistant. In *Proceedings of the 2016 12th IEEE/ASME International Conference on Mechatronic and Embedded Systems and Applications (MESA)* 29–31 (2016).

[6] Shen, B., Zhang, W. Y., Qi, D. P. & Wu, X. Y. Wireless multimedia sensor network based subway tunnel crack detection method. *Int. J. Distrib. Sens. Netw.* **11** (6), 184639 (2015).

[7] Menendez, E., Victores, J., Montero, R., Martinez, S. & Balaguer Carlos. Tunnel structural inspection and assessment using an autonomous robotic system. *Autom. Construct.*. **87** 117–126. (2018).

[8] Huang, H., Sun, Y., Xue, Y. & Wang, F. Inspection equipment study for subway tunnel defects by grey-scale image processing. *Adv. Eng. Inf. Volume*. **32**, 188–201 (2017).

[9] Idoux, M. Multisensor system for tunnel inspection. *Proc. SPIE-The Int. Soc. Opt. Eng.* **5640**, 303–312 (2005).

[10] Lee, B. Y. et al. Automated image processing technique for detecting and analysing concrete surface cracks. *Struct. Infrastruct. Eng.* **9** (6), 567–577 (2013).

[11] Xiaojun, W. U., Shaohong, B. A., Binggiang, C. H. U. A. I., Rongchang, C. H. E. N. & Pu, J. I. A. N. G. Fast detection system for metro tunnel crack based on CMOS Line-scan digital camera. *Subgrade Eng.* (03), 185–190. (2015).

[12] Müller, U., Kuhn, P. & Kadner, G. *tCrack Automatic Crack Detection in Tunnels* (World Tunneling, 2012).

[13] ZHOU Weizhen. *Research on Tunnel Image Acquisition System* (Beijing Jiaotong University, 2021).

[14] LIU Xuanran. *Tunnel Crack Image Acquisition and Detection Technology Based on Area Scan Camera* (Beijing Jiaotong University, 2019).

[15] Hongwei Huang, S., Zhao, D., Zhang, J. & Chen Deep learning-based instance segmentation of cracks from shield tunnel lining images. *Struct. Infrastruct. Eng.* (2020).

[16] Lingzhi, Y. A. N. G. & Enquan, F. A. N. G. Review and developing trend on technology for detecting metro tunnel structure diseases. *Urban Rapid Rail Transit.* **30** (01), 20–25 (2017).

[17] Sun, H. et al. Tunnel monitoring and measuring system using mobile laser scanning: design and deployment. *Remote Sens.* **12**, 730 (2020).

[18] Bar-Itzhack, I. Y. & Berman, N. Control theoretic approach to inertial navigation systems. *Ournal Guidance Control Dynamics*. **11** (3), 237–245 (1988).

[19] Li, Z., Wang, J., Li, B., Gao, J. & Tan, X. GPS/INS/Odometer integrated system using fuzzy neural network for land vehicle navigation applications. *J. Navig.* **67** (6), 967–983 (2014).

[20] Wang, W. & Wang, D. September. Land vehicle navigation using odometry/INS/vision integrated system. In *Proceedings of the 2008 IEEE Conference on Cybernetics and Intelligent Systems* **21–24** 754–759. (2008).

[21] Trimble, M. Dead reckoning. *CNS Spectr.* **7**, 565 (2002). 15094691 10.1017/s1092852900018150

[22] Shin, E. H. Accuracy Improvement of Low Cost INS/GPS for Land Applications. Master’s Thesis, The University of Calgary. (2001).

[23] Du, L., Zhong, R., Sun, H. & Wu, Q. Automatic monitoring of tunnel deformation based on high density point clouds data. *Int. Archives Photogrammetry Remote Sens. Spat. Inform. Sci.* **42**, 353–360 (2017).

[24] Kang, Z., Tuo, L. & Zlatanova, S. Continuously deformation monitoring of subway tunnel based on terrestrial point clouds. *Int. Archives Photogrammetry Remote Sens. Spat. Inform. Sci.* **39**, 199–203 (2012).

[25] Tuo, L. et al. Continuously vertical section abstraction for deformation monitoring of subway tunnel based on terrestrial point clouds. *Geomatics Inform. Sci. Wuhan Univ.* **38** (2), 171–175185 (2013).

[26] Kang, Z. Z., Zhang, L. Q., Tuo, L., Wang, B. & Chen, J. Continuous extraction of subway tunnel cross sections based on terrestrial point clouds. *Remote Sens.* **6**, 857–879 (2014).

[27] Yang, S. L., Liu, W. N., Wang, M. S., Huang, F. & Cui, N. Z. Study on the auto-total station system for monitoring analyzing and forecasting tunnel country rock deformation. *J. China Railw Soc.* **26**, 93–97 (2004).

[28] Montero, R. et al. The robot-spect eu project: Autonomous robotic tunnel inspection. Robocity2030 13th Workshop EU robotic projects results. 91–100. (2015).

[29] Tang, C. et al. Studies on automatic extraction of precise 3D laser measurement data in specific section of subway tunnel. *Proc. SPIE 11562, AOPC 2020: Advanced Laser Technology and Application* 1156212. (2020).

[30] Engstrand, A. Railway surveying-a case study of the GRP 5000. (2011).

[31] Leica SiTrack. One track maintenance and renovation solution. (Accessed 9 December 2019). http://www.leica-geosystems.com.cn/leica_geosystems

[32] Huang, H. et al. Inspection equipment study for subway tunnel defects by grey-scale image processing. *Adv. Eng. Inform.* **32**, 188–201 (2017).

[33] TS4 recording system. https://www.spacetec.de/en/products/ts4/

[34] Menendez, E. et al. Tunnel structural inspection and assessment using an autonomous robotic system. *Autom. Constr.* **87**, 117–126 (2018).

[35] Huang, H., Zhao, S., Zhang, D. & Chen, J. Deep learning-based instance segmentation of cracks from shield tunnel lining images. *Struct. Infrastruct. Eng.* **18** (2), 183–196 (2022).

